# 203. Activity of Cefepime in Combination with Taniborbactam (formerly VNRX-5133) Against *Pseudomonas aeruginosa* from a Global 2018-2020 Surveillance Collection

**DOI:** 10.1093/ofid/ofab466.203

**Published:** 2021-12-04

**Authors:** Meredith Hackel, Mark G G Wise, Daniel F Sahm

**Affiliations:** 1 IHMA, Inc., Schaumburg, Illinois; 2 IHMA, Schaumburg, Illinois

## Abstract

**Background:**

Taniborbactam is a novel cyclic boronate-based broad-spectrum β-lactamase inhibitor (BLI) with potent and selective inhibitory activity against both serine- and metallo-β-lactamases (MBLs). Taniborbactam restores the activity of cefepime (FEP) against many multidrug resistant organisms, including cephalosporin- and carbapenem-resistant Enterobacterales and *Pseudomonas aeruginosa* (PA). We evaluated the *in vitro* activity of the investigational combination cefepime-taniborbactam and comparators against clinical isolates of PA collected during a 2018-2020 surveillance.

**Methods:**

MICs of FEP with taniborbactam fixed at 4 µg/mL (FTB) and comparators were determined against 3,219 PA collected from 221 sites in 52 countries in 2018-2020. Resistant phenotypes were based on 2021 CLSI breakpoints. Acquired β-lactamase (BL) genes were identified via PCR/Sanger sequencing or whole-genome sequencing (WGS) for 516 isolates with meropenem (MEM) MIC ≥8 µg/mL, and for 94 randomly selected isolates with FEP or ceftazidime MIC ≥16 µg/mL. 186 isolates with FTB MIC ≥16 µg/mL, 16 with FTB MIC=8 µg/mL and one with FTB MIC=4 µg/mL were subjected to WGS.

**Results:**

Overall, 28.7%, 26.2% and 20.3% of PA isolates were nonsusceptible (NS) to piperacillin-tazobactam (TZP), MEM or FEP, respectively (Table). FTB demonstrated potent activity (MIC_50/90_, 2/8 µg/mL; 94.2% inhibited at ≤8 µg/mL) against PA overall and inhibited between 63.4% (ceftazidime-avibactam [CZA] NS) and 82.1% (TZP NS) of isolates in the NS subsets compared to 0% to 69.1% S for comparators. Against the 111 strains carrying VIM or NDM MBL genes, 67.6% had FTB MICs ≤8 µg/mL, with 11.7% having FTB MICs of 16 µg/mL. Plausible explanations for elevated FTB MICs included IMP MBL genes, penicillin binding protein 3 variations, and/or possible efflux pump up-regulation.

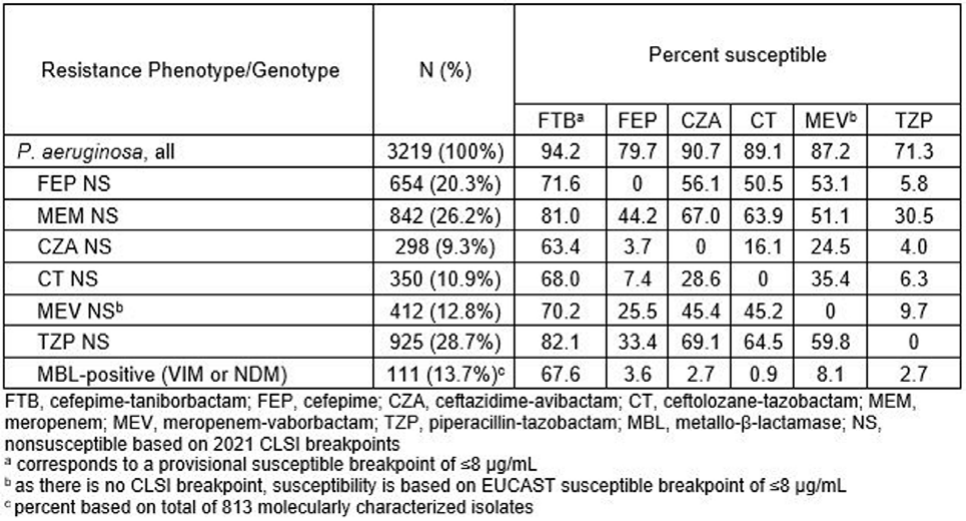

**Conclusion:**

FTB demonstrated potent *in vitro* activity against PA with different resistance profiles, including NS to FEP, MEM, and TZP, and to the BL/BLI combinations CZA, ceftolozane-tazobactam, and meropenem-vaborbactam. FTB was the most active agent tested against PA harboring VIM and NDM MBLs. These findings support the continued development of FTB as a potential new treatment option for challenging infections due to MDR PA.

**Disclosures:**

**Meredith Hackel, PhD MPH**, **IHMA** (Employee)**Pfizer, Inc.** (Independent Contractor) **Mark G G. Wise, PhD**, **IHMA** (Employee)**Pfizer, Inc.** (Independent Contractor) **Daniel F. Sahm, PhD**, **IHMA** (Employee)**Pfizer, Inc.** (Independent Contractor)

